# Supplementary figures and images for: Impact of preventive chemotherapy on Strongyloides stercoralis: A systematic review and meta-analysis
Source: PLoS Negl Trop Dis. 2023 Jul 10;17(7):e0011473. doi: 10.1371/journal.pntd.0011473 (PMC10358935; doi:10.1371/journal.pntd.0011473)

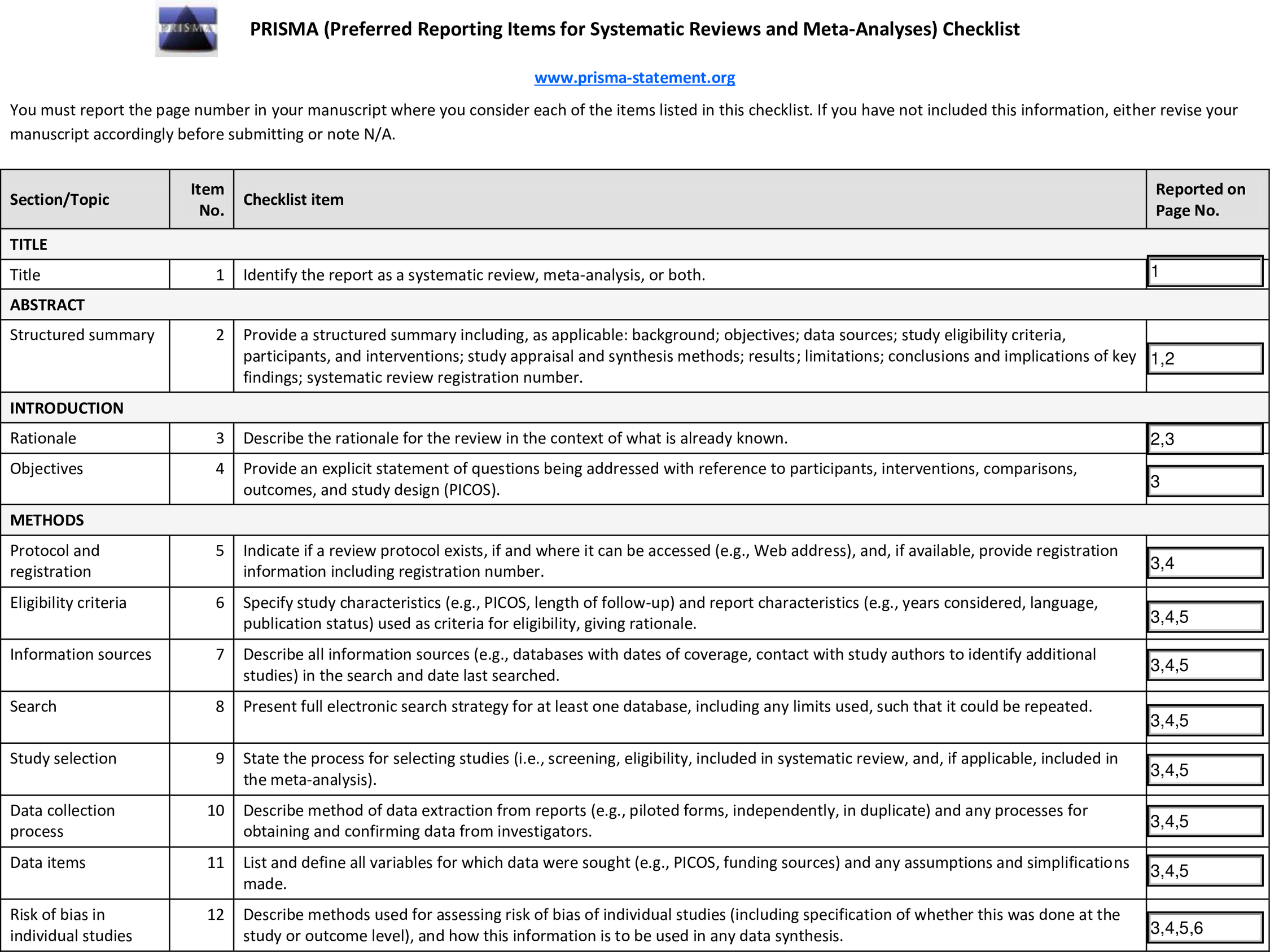

Supplement: S1 File — (TIF) [file pntd.0011473.s001.tif]
