# Supplementary material for: Impact of preventive chemotherapy on Strongyloides stercoralis: A systematic review and meta-analysis
Source: PLoS Negl Trop Dis. 2023 Jul 10;17(7):e0011473. doi: 10.1371/journal.pntd.0011473 (PMC10358935; doi:10.1371/journal.pntd.0011473)
Supplement: S2 File — (PDF) [file pntd.0011473.s002.pdf]

NEWCASTLE - OTTAWA QUALITY ASSESSMENT SCALE
COHORT STUDIES

Selection

1) Representativeness of the exposed cohort

a) MDA intervention or cohort intervention study on whole population (randomized or voluntary participation)*

b) MDA intervention or cohort intervention study on selected broad population groups (e.g. SAC)*

c) MDA intervention or cohort intervention study on selected restricted population groups (e.g. pregnant women)

d) no description of the derivation of the cohort

2) Study design

a) prospective *

b) retrospective

c) no description

3) Ascertainment of infection

a) Serology/Baermann/APC/PCR*

c) Kato-Katz or FECT

d) no description

4) Data availability

a) Full text_ all data reported *

b) Abstract

c) Full text or abstract_ incomplete data reporting

Comparability

1) Cohort of controls

a) Present*

b) Absent

2) Same diagnostic method for baseline and follow-up infection assessment

a) Yes *

b) No

Outcome

1) Assessment of outcome

a) Independent blind assessment*

b) No blinding

c) No description

2) Was follow-up long enough for outcomes to occur

a) Yes (at least 12 months for follow up after the last treatment)*
b) No

3) Adequacy of follow up of cohorts

a) total population examined at the last follow up time point after treatment >=80% of baseline*
b) total population examined at the last follow up time point after treatment <80% of baseline
d) follow up only of individuals positive at baseline (or previous follow up time point) only
d) no statement
