# Supplementary material for: Impact of preventive chemotherapy on Strongyloides stercoralis: A systematic review and meta-analysis
Source: PLoS Negl Trop Dis. 2023 Jul 10;17(7):e0011473. doi: 10.1371/journal.pntd.0011473 (PMC10358935; doi:10.1371/journal.pntd.0011473)
Supplement: Attachment — Submitted filename: response to reviewers.docx [file pntd.0011473.s004.docx]

Dear Editor,

Please find here below our point-by-point reply to the reviewers’ comments. We are grateful for their thorough revision that permitted to improve our manuscript.

We wish to highlight that, as you can read in some of our answers, we aimed to follow the systematic review methodology rigorously, in order to obtain robust and meaningful results, as we believe we did.

Reviewer #1: The objective of the review and meta-analysis was clearly articulated, and the study design was appropriate to address this.
The inclusion and exclusion criteria for studies included in the review and meta-analysis were clearly stated and were appropriate for the study.
The sample size, as shown by the small number of studies included in the review meta-analysis, was not sufficient. However, this limitation was beyond the authors, as data which accounted for the limitation, were not available in included studies. This limitation was stated by the authors in their conclusion.  
The conclusions were backed up by correct statistical analyses
There are no concerns about ethical or regulatory requirements being met for the conduct of the study

**Authors. We thank the reviewer for this positive feedback on study design and methods**

Reviewer #2: -Are the objectives of the study clearly articulated with a clear testable hypothesis stated? The goal of the paper should be reviewed since there is not enough information in the literature to actually support the intention of the paper.

**Authors. We agree that the information retrieved by the literature is scarce. However, systematic reviews have rigorous methods to be followed, and these include the publication of a study protocol that should be registered and carefully assessed *a priori*. Changing study objectives and methods once the review is completed would mean to start with a novel systematic review. Moreover, as pointed out by reviewer 1, the limited number of studies is a limitation beyond our capacities, and we stated this in the discussion. Then please consider that despite the limited number of studies, the heterogeneity (assessed by I^2^) for the main outcome was low, demonstrating robust results. Finally, this review aimed to support specifically the work which is currently underway for the production of WHO guidelines on control of strongyloidiasis, with the current state of knowledge about the topic.**

-Is the study design appropriate to address the stated objectives? Yes
-Is the population clearly described and appropriate for the hypothesis being tested? Yes
-Is the sample size sufficient to ensure adequate power to address the hypothesis being tested? No

**Authors. As stated in our previous comment, I^2^ statistics supported the robustness of the results of our primary outcome. According to systematic review methodology, two studies is the minimum number for meta-analysis (see Cochrane for systematic reviews, chapter 10, available at:** [**https://training.cochrane.org/handbook/current/chapter-10**](https://training.cochrane.org/handbook/current/chapter-10)**). We had six studies based on fecal-based diagnostic methods and three based on serology diagnostic methods to explore our primary outcome, and both analyses had low heterogeneity. Heterogenity was 0 for the analysis based on fecal tests and around 4 (which is also extremely low) for studies based on serology. Hence, we do not understand which is the parameter considered by the reviewer to state that the sample size was not sufficient for our primary outcome. Please let us know if this consideration is based on the guidelines for systematic reviews.**

-Were correct statistical analysis used to support conclusions? Yes
-Are there concerns about ethical or regulatory requirements being met? No

Reviewer #3: Methods employed are adequate
--------------------

<b>Results</
-Does the analysis presented match the analysis plan?</br>
-Are the results clearly and completely presented?</br>
-Are the figures (Tables, Images) of sufficient quality for clarity?</br></br>

Reviewer #1: The analysis presented match the analysis plan.
The results were clearly presented.
However,:
1. Year of publication should follow each of the author names for all studies listed under the column Study" in Table 1.
2. et al. should follow each of the author names listed under the column "Study" in Figures 2, 3, 4 & 5.

**Authors. Thanks for your comments, we revised Table 1 accordingly.**

Reviewer #2: -Does the analysis presented match the analysis plan? Yes
-Are the results clearly and completely presented? In part. There is a substantial amount of information in the papers that they use as a reference that could be explored to increase the relevance of the review.

**Authors. We thank the reviewer for this comment. We presented the main information retrieved from the papers in Table 1, but we understand that not all aspects/details might be included there. Hence, we tried to improve the text with some additional information that we deem worth of note(lines 173-183 and 215-219, clean version of reviewed manuscript). However, we tried to limit focusing on data that cannot be analyzed formally, as their impact is weaker compared to data that can be meta-analyzed.**

-Are the figures (Tables, Images) of sufficient quality for clarity? Yes

Reviewer #3: Results are adequately presented and dicussed.

**Authors. Thanks**

--------------------

<b>Conclusions</b></br></br>
-Are the conclusions supported by the data presented?</br>
-Are the limitations of analysis clearly described?</br>
-Do the authors discuss how these data can be helpful to advance our understanding of the topic under study?</br>
-Is public health relevance addressed?</br></br>

Reviewer #1: The conclusions are justified by the data presented in the manuscript.
The limitations of the analysis were described, although not in the conclusion, but rather in the "Discussion" section.
The authors did discuss how data from their study could be helpful to advance our understanding of the subject matter under study as well as the public health relevance of their study/analysis. These were captured in the "Discussion" section.

**Authors. We thank the reviewer for the positive feedback**

Reviewer #2: -Are the conclusions supported by the data presented? Yes, but since the authors don't have enough information to support their main goal, the conclusion is scarce.

**Authors. We thank the reviewer for pointing out the consistency between findings and conclusions. Again, we wish to highlight the fact that we followed the rigorous guidelines for systematic reviews, and we believe that the results of our primary outcome are robust in spite of the limited number of papers included.**

-Are the limitations of analysis clearly described? Yes
-Do the authors discuss how these data can be helpful to advance our understanding of the topic under study? Yes
-Is public health relevance addressed? Yes

Additional comment: The paragraph were the authors discuss the use of a combination of anthelminthics is not helpful to prove their point, and makes the reader wonder why they don't did the review also based in other possible treatments.

**Authors. We are grateful to the reviewer for this meaningful comment. We understand that the paragraph can be misleading, hence we deleted it**

Reviewer #3: Conclusions are adequately formulated and limitations in the analysis are clearly detailed. The public health implications of the findings are addressed.

**Authors. We thank the reviewer for this positive feedback**

--------------------

<b>Editorial and Data Presentation Modifications?</b></br><br/>
Use  this section for editorial suggestions as well as relatively minor modifications of existing data that would enhance clarity. If the only modifications needed are minor and/or editorial, you may wish to recommend “Minor Revision” or “Accept”.

Reviewer #1: Minor Revision; mainly editorial.
Please see attached "Reviewed" manuscript for details.

**Authors. We are grateful for the thorough revision of the manuscript, which allowed us to revise the manuscript based on the reviewer’s annotated document and improve it throughout.**

**.**
Reviewer #2: (No Response)

Reviewer #3: Reference [3] is not the WHO road map for NTDs, although the text on lines 49-51 seems to imply this. You may wish to add the road map as appropriate.

Please spell "roadmap" as "road map" (two words).

**Authors. Thanks for noticing the wrong reference, we have fixed it. Also, we amended the word “road map”**

--------------------

<b>Summary and General Comments</b></br></br>
Use  this section to provide overall comments, discuss strengths/weaknesses of the study, novelty, significance, general execution and scholarship. You may also include additional comments for the author, including concerns about dual publication, research ethics, or publication ethics. If requesting major revision, please articulate the new experiments that are needed.

Reviewer #1: The conceptualization and execution of the study was adequate.
The "References" section needs to be adequately revised. Please see attached "Reviewed" manuscript.

**Authors. Again, many thanks for providing an edited version of the manuscript**

Reviewer #2: As addressed by the authors in the discussion there is not enough data in the literature that support this review. With that, there is no clear conclusion and no addition of information to the knowledge that the scientific community already has.
**Authors. As we commented previously, we rigorously followed the systematic reviews guidelines. Based on these, we can state that our results are meaningful despite the small number of papers included, and we could provide conclusions, of course based on the available evidence. Moreover, it is well appreciated that systematic reviews are useful also to highlight the need for further evidence on a specific topic. This is something that emerges here, and we discuss this issue in the Discussion.**

**Finally, the study primary objective is of relevance for the upcoming WHO guidelines for the control of strongyloidiasis in endemic areas (two of the authors of this review are members of the WHO Strongyloides guideline working group): although randomized controlled trials demonstrated the efficacy of ivermectin for individual treatment of strongyloidiasis, evidence on community treatment is also needed, because additional factors might contribute to affect the intervention. Therefore, a systematic review on this topic was timely and needed to support WHO activities.**

Reviewer #3: An interesting review assessing the impact of preventive chemotherapy (PC) with ivermectin on prevalence of S. stercoralis infection in endemic areas, with the aim of generating evidence that can inform global health policy.

In spite of the limited number of studies included in the analysis and the heterogeneity of such studies in terms of target  population, frequency of administration of IVM and follow-up delay, results are consistently supporting MDA with IVM as a key public health intervention against Strongyloides stercoralis.

I don't have any specific technical comment on the article.

**Authors. We thank the reviewer for the thoughtful positive comment.**
